# Draft Genome Sequence of *Bacillus* sp. Strain CDB3, an Arsenic-Resistant Soil Bacterium Isolated from Cattle Dip Sites

**DOI:** 10.1128/genomeA.00429-17

**Published:** 2017-06-22

**Authors:** Yiren Yang, Ren Zhang

**Affiliations:** School of Biological Sciences, University of Wollongong, Wollongong, New South Wales, Australia

## Abstract

*Bacillus* sp. strain CDB3, isolated from cattle dip sites in Australia, is highly resistant to arsenic. It contains 22 arsenic resistance (*ars*) genes, of which 17 are organized in 3 *ars* clusters. Here, we report the draft genome sequence of CDB3, which will assist us in understanding the overall *ars* mechanism.

## GENOME ANNOUNCEMENT

Arsenic contamination in soil and water is a global issue. Microbes that possess high arsenic resistance (*ars*) can provide insights into the resistance mechanism and may assist with treating arsenic poisoning. *Bacillus* sp. strain CDB3 was isolated from arsenic-contaminated cattle tick dip sites in Australia and was previously identified based on its 16S classification and fatty acid composition profile (1). It demonstrated a significantly high tolerance toward arsenic (1), and two *ars* clusters were already identified (2). *Bacillus* sp. CDB3 serves a good system for *ars* study, possessing not only novel* ars* genes but also, interestingly, a rare *ars* cluster (2).

Genomic DNA library construction, pyrosequencing, data assembly, and annotation were carried out as previously described (3). Draft genome sequences were also submitted to PHASTER for prophage identification (4).

The draft genome size of *Bacillus* sp. CDB3 comprises approximately 6,000 kb, with a coverage of about 45-fold. The assembled genome contained 148 contigs (*N*_50_ value, 104,943 bp) arranged into 19 scaffolds (*N*_50_ value, 4,125,929 bp), with a G+C content of 35.3%. There were 6,236 coding sequences (CDSs; length >100 bp) and 115 RNAs found in the genome, while the closest neighbor based on genome comparison was *Bacillus cereus* BAG1X1-3 (accession no. GCA_000291055.1). An intact prophage (phIS3501, accession no. NC_019502) was also found in the genome.

Genome analysis of CDB3 confirmed the 2 *ars* gene clusters found in our previous study (2). The long 8-gene operon, *ars1* (*arsRYCDATorf7orf8*), possesses an interesting posttranscriptional regulation mechanism (5) and 2 novel genes, *orf7* and *orf8*, coding for proteins homologous to HesB and dual-specificity protein phosphatase, respectively (6). The second operon, *ars2,* is highly homologous to other *arsRorf2YC* operons which are present in the *skin* element (7). However, as an intact *sigK* was found, *ars2* was suggested not to be part of the *skin* element in our strain. Note that an *orf2* homologue has been found to be responsible for MAs(III) demethylation and was denoted *arsI* (8). Furthermore, an additional *ars* cluster (*ars3*) was revealed upstream of *ars1*, harboring 5 genes that were related to arsenic resistance (*arsR_3_TO_1_O_2_N*). Although *ars1* and *ars3* were found located relatively close (apart by 7,046 bp) on a 24,354-bp mobile genomic island, our previous study based on resistance screening did not isolate *ars3* (2), suggesting that this cluster alone may not confer significant arsenic resistance.

Furthermore, several individually located *ars* gene homologues were also found in the CDB3 genome, including 4 additional arsenite transporters (ArsY3 and 3 ArsBs) and one other ATPase (ArsA2). The draft genome sequence of CDB3 provides further information for understanding arsenic resistance in bacteria.

### Accession number(s).

This whole-genome shotgun project has been deposited at DDBJ/EMBL/GenBank under the accession no. ALBR00000000. The version described in this paper is the first version, ALBR01000000.
